# Spectral pruning of fully connected layers

**DOI:** 10.1038/s41598-022-14805-7

**Published:** 2022-07-01

**Authors:** Lorenzo Buffoni, Enrico Civitelli, Lorenzo Giambagli, Lorenzo Chicchi, Duccio Fanelli

**Affiliations:** 1 Instituto de Telecomunicações, Physics of Information and Quantum Technologies Group, Lisbon, Portugal; 2 CSDC, Department of Physics and Astronomy, University of Florence, Sesto Fiorentino, Italy; 3 LabGOL, Department of Information Engineering, University of Florence, Florence, Italy; 4 naXys-Namur Center for Complex Systems, University of Namur, rue Grafé 2, 5000 Namur, Belgium

**Keywords:** Computer science, Complex networks, Information theory and computation

## Abstract

Training of neural networks can be reformulated in spectral space, by allowing eigenvalues and eigenvectors of the network to act as target of the optimization instead of the individual weights. Working in this setting, we show that the eigenvalues can be used to rank the nodes’ importance within the ensemble. Indeed, we will prove that sorting the nodes based on their associated eigenvalues, enables effective pre- and post-processing pruning strategies to yield massively compacted networks (in terms of the number of composing neurons) with virtually unchanged performance. The proposed methods are tested for different architectures, with just a single or multiple hidden layers, and against distinct classification tasks of general interest.

## Introduction

Automated learning via deep neural networks is gaining increasing popularity, as a ductile procedure to address a widespread plethora of interdisciplinary applications^1–3^. In standard neural network training one seeks to optimise the weights that link pairs of neurons belonging to adjacent layers of the selected architecture^4^. This is achieved by computing the gradient of the loss with respect to the sought weights, a procedure which amounts to operate in the so called direct space of the network^5^. Alternatively, the learning can be carried out in reciprocal space: the spectral attributes (eigenvalues and eigenvectors) of the transfer operators that underlie information handling across layers define the actual target of the optimisation. This procedure, first introduced in^5^ and further refined in^6^, enables a substantial compression of the space of trainable parameters. The spectral method leverages on a limited subset of key parameters which impact on the whole set of weights in direct space. Particularly relevant, in this respect, is the setting where the eigenmodes of the inter-layer transfer operators align along random directions. In this case, the associated eigenvalues constitute the sole trainable parameters. When employed for classifications tasks, the accuracy displayed by the spectral scheme restricted to operate with eigenvalues is slightly worse than that reported when the learning is carried in direct space, for an identical architecture and by employing the full set of trainable parameters. To bridge the gap between conventional and spectral methods in terms of measured performances, one can also train the elements that populate the non trivial block of the eigenvectors matrix^5^. By resorting to apt decomposition schemes, it is still possible to contain the total number of trainable parameters, while reaching stunning performances in terms of classification outcomes^6^.

In this paper we will discuss a relevant byproduct of the spectral learning scheme. More specifically, we will argue that the eigenvalues do provide a reliable ranking of the nodes, in terms of their associated contribution to the overall performance of the trained network. Working along these lines, we will empirically prove that the absolute value of the eigenvalues is an excellent marker of the node’s significance in carrying out the assigned discrimination task. This observation can be effectively exploited, downstream of training, to filter the nodes in terms of their relative importance and prune the unessential units so as to yield a more compact model, with almost identical classification abilities. The effectiveness of the proposed method has been tested for different feed-forward architectures, with just a single or multiple hidden layers, by invoking several activation functions, and against distinct datasets for image recognition, with various levels of inherent complexity. Building on these findings, we will also propose a two stages training protocol to generate minimal networks (in terms of allowed computing neurons) which outperform those obtained by hacking off dispensable units from a large, fully trained, apparatus. This strategy can be seen as an effective way to discover sub-networks (a.k.a. “winning tickets”^7^) with recorded performance comparable to those displayed by their unaltered homologues, after a proper round of training^7^. More specifically, after a first round of training which solely acts on the eigenvalues, one can identify the most relevant nodes, as follows the magnitude of the associated eigenvalues. Since the first training stage is just targeted to eigenvalues, the eigenvectors obtained after pruning are still bearing reflexes of the random initialization and thus represent a sort of “winning ticket”^7^. In this respect, according to the above reasoning, the proposed two stages strategy can be seen as a novel and efficient way to discover optimal sub-networks.

The paper is organized as follows. In the next section we will discuss the mathematical foundation and set the notation of the spectral learning scheme. We will then move on to illustrating the results of the proposed spectral pruning strategy, after a short account of the alternative methods available in the literature. Finally, we will sum up and draw our conclusions. The details about the proposed schemes are discussed in “Methods” section.

## Spectral approach to learning

This Section is devoted to reviewing the spectral approach to the training of deep neural networks. The discussion will follow mainly^6^, where an extension of the method originally introduced in^5^ is handed over. For the sake of completeness, let us emphasize a substantial difference between these works^5,6^ and the one proposed in this manuscript: in Giambagli et al.^5^ and Chicchi et al.^6^ the focus is on designing a training algorithm in the spectral domain while, in this work, we propose a novel idea to effectively prune fully connected layers by exploiting the spectral approach to neural network training.

Consider a deep feed-forward network made of $\ell$ distinct layers. Each layer is labelled with a discrete index *i* $(=1,\ldots ,\ell )$. Denote by $N_i$ the number of the neurons, the individual computing units, that pertain to layer *i*. Then, we posit $N=\sum _{i=1}^{\ell } N_i$ and introduce a column vector $\vec {x}^{(1)}$, of size *N*, the first $N_1$ entries referring to the supplied input signal. As anticipated, we will be mainly concerned with datasets for image recognition, so we will use this specific case to illustrate the more general approach of spectral learning. This means that, the first $N_1$ elements of $\vec {x}^{(1)}$ are the intensities (from the top-left to the bottom-right, moving horizontally) as displayed on the pixels of the image presented as an input. All other entries of $\vec {x}^{(1)}$ are identically equal to zero.

**Figure 1:**
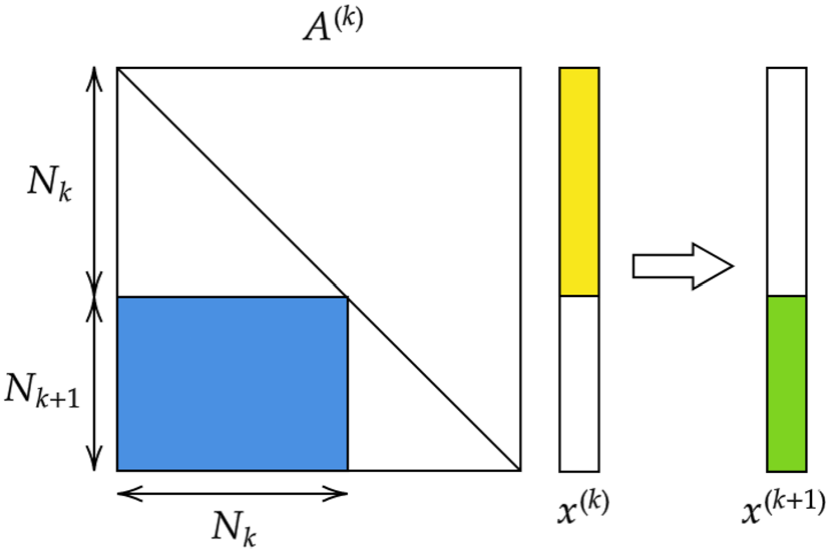
A schematic outline of the structure of transfer matrix ${{\varvec{A}}}^{(k)}$, bridging layer *k* to layer $k+1$. The action of ${{\varvec{A}}}^{(k)}$ on $\vec {x}^{(k)}$ is also graphically illustrated.

**Figure 2:**
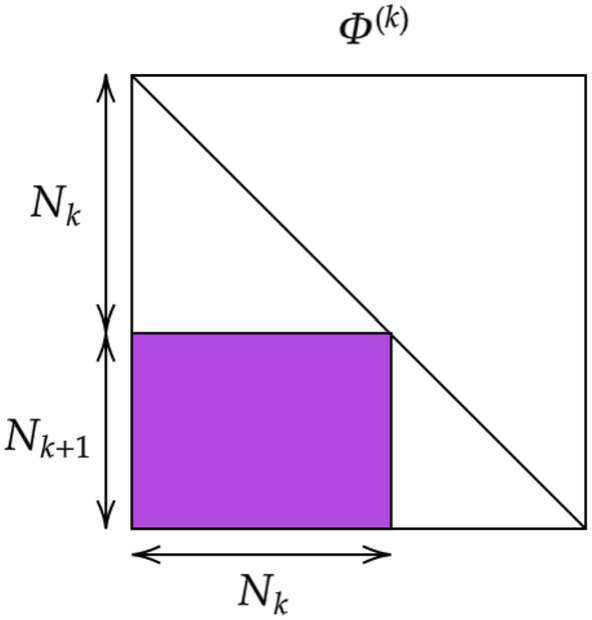
The structure of matrix ${\varvec{\Phi }}^{(k)}$ is schematically displayed.

The aim of the procedure is to map $\vec {x}^{(1)}$ into an output vector $\vec {x}^{(\ell )}$, still of size *N*: the last $N_{\ell }$ elements are the intensities displayed at the output nodes, where reading is eventually performed. The applied transformation is composed by a suite of linear operations, interposed to non linear filters. To exemplify the overall strategy, consider the generic vector $\vec {x}^{(k)}$, with $k=1,\ldots , \ell -1$, as obtained after *k* execution of the above procedure. At the successive iteration, one gets $\vec {x}^{(k+1)}= {{\varvec{A}}}^{(k)} \vec {x}_{(k)}$, where ${{\varvec{A}}}^{(k)}$ is a $N \times N$ matrix with a rather specific structure, as elucidated in the following and schematically depicted in Fig. 1. Further, a suitably defined non-linear function $f(\cdot , \beta _k)$ is applied to $\vec {x}^{(k+1)}$, where $\beta _k$ identifies an optional bias. To proceed in the analysis, we cast ${{\varvec{A}}}^{(k)}={{\varvec{\Phi }}}^{(k)} {{\varvec{\Lambda }}}^{(k)} ({{\varvec{\Phi }}}^{(k)})^{-1}$ by invoking spectral decomposition. Here, ${{\varvec{\Lambda }}}^{(k)}$ denotes the diagonal matrix of the eigenvalues of ${{\varvec{A}}}^{(k)}$. Following^6^, we set $({{\varvec{\Lambda }}}^{(k)} )_{jj} = 1$ for $j< \sum _{i=1}^{k-1} N_i$ and $j> \sum _{i=1}^{k+1} N_i$. The remaining $N_k+N_{k+1}$ elements are initially assigned to random entries, as e.g. extracted from a uniform distribution, and define a first basin of target variables for the spectral learning scheme. Then, ${{\varvec{\Phi }}}^{(k)}$ is the identity matrix $\mathbb {I}{}_{N \times N}$, with the inclusion of a sub-diagonal $N_{k+1} \times N_{k}$ block, denoted by ${\varvec{\phi }}^{(k)}$, see Fig. 2. This choice amounts to assume a feed-forward architecture. It can be easily shown that $({{\varvec{\Phi }}}^{(k)})^{-1}=2 \mathbb {I}{}_{N \times N}- {{\varvec{\Phi }}}^{(k)}$, which readily yields ${{\varvec{A}}}^{(k)}={{\varvec{\Phi }}}^{(k)} {{\varvec{\Lambda }}}^{(k)} (2 \mathbb {I}{}_{N \times N}- {{\varvec{\Phi }}}^{(k)} )$. The off-diagonal elements of ${{\varvec{\Phi }}}^{(k)}$ define a second set of adjustable parameters to be self-consistently modulated during active training. To implement the learning scheme on these basis, we consider $\vec {x}^{(\ell )}$, the image on the output layer of the input vector $\vec {x}^{(1)}$:

1 $$\begin{aligned} \vec {x}^{(\ell )} = f\left( {\varvec{A}}^{(\ell -1)}\ldots f\left( {\varvec{A}}^{(1)} \vec {x}^{(1)},\beta _1 \right) ,\beta _{\ell -1} \right) \end{aligned}$$

Since we are dealing with image classification, we can calculate $\vec {z} = softmax(\vec {x}^{(\ell )})$. We will then use $\vec {z}$ to compute the categorical cross-entropy loss function $\text {CCE}(l(\vec {x}^{(1)}), \vec {z})$, where $l(\vec {x}^{(1)})$ is the label which identifies the category to which $\vec {x}^{(1)}$ belongs, via one-hot encoding^8^.

The loss function can thus be minimized by acting on the spectral parameters, i.e. the ensemble made of non trivial eigenvalues and/or the associated eigendirections. A straightforward calculation, carried out in the annexed  Supplementary Information, allows one to derive a closed analytical expression for $w^{(k)}_{ij}$, the weights of the edges linking nodes *i* (belonging to layer $k+1$) and *j* (sitting on layer *k*) in direct space, as a function of the underlying spectral quantities. In formulae, one gets:

2 $$\begin{aligned} w^{(k)}_{ij} = \left( \lambda ^{(k)}_{m(j)}-\lambda ^{(k)}_{l(i)} \right) {\Phi }^{(k)}_{l(i), m(j)} \end{aligned}$$

where $l(i)=\sum _{s=1}^{k} N_s +i$ and $m(j)=\sum _{s=1}^{k-1} N_s +j$, with $i \in \left( 1, \ldots , N_{k+1} \right)$ and $j \in \left( 1, \ldots , N_k \right)$. In the above expression, $\lambda ^{(k)}_{m(j)}$ stand for the first $N_k$ eigenvalues of ${{\varvec{\Lambda }}}^{(k)}$. The remaining $N_{k+1}$ eigenvalues are labelled $\lambda ^{(k)}_{l(i)}$.

To help comprehension denote by $x^{(k)}_j$ the activity on nodes *j*. Then, the activity $x^{(k)}_i$ on node *i* reads:

3 $$\begin{aligned} \tiny {x^{(k+1)}_i = \sum _{j=1}^{N_k} \left( \lambda ^{(k)}_{m(j)} {\Phi }^{(k)}_{l(i), m(j)} x_j^{(k)} \right) - \lambda ^{(k)}_{l(i)} \sum _{j=1}^{N_k} \left( {\Phi }^{(k)}_{l(i), m(j)} x_j^{(k)} \right) } \end{aligned}$$

The eigenvalues $\lambda ^{(k)}_{m(j)}$ modulate the density at the origin, while $\lambda ^{(k)}_{l(i)}$ set the excitability of the receiver nodes, weighting the network activity in its immediate neighbourhood. As remarked in^6^, this can be rationalized as the artificial analogue of the *homeostatic plasticity*, the strategy used by living neurons to maintain the synaptic basis for learning, respiration, and locomotion^9^.

Starting from this background, we shall hereafter operate within a simplified setting which is obtained by imposing $\lambda ^{(k)}_{m(j)} = 0$. This implies that $\lambda ^{(k)}_{l(i)}$ are the sole eigenvalues to be actively involved in the training. As we shall prove, these latter eigenvalues provide an effective criterion to rank a posteriori, i.e. upon training being completed, the relative importance of the nodes belonging to the examined network. Stated differently, nodes can be sorted according to their relevance in carrying out the assigned task. This motivates us to introduce, and thoroughly test, an effective spectral pruning strategy which seeks at removing the nodes deemed unessential, while preserving the overall network classification score. “Methods” is entirely devoted to explain in detail the proposed strategy, that we shall contextualize with reference to other existing methodologies.

## Conventional pruning techniques

Generally speaking, it is possible to ideally group various approaches for network compression into five different categories: Weights Sharing, Network Pruning, Knowledge Distillation, Matrix Decomposition and Quantization^10,11^.

Weights Sharing defines one of the simplest strategies to reduce the number of parameters, while allowing for a robust feature detection. The key idea is to have a shared set of model parameters between layers, a choice which reflects back in an effective model compression. An immediate example of this methodology are the convolutional neural networks^12^. A refined approach is proposed in Bat et al.^13^ where a virtual infinitely deep neural network is considered. Further, in Zhang et al.^14^ an $\ell _{1}$ group regularizer is exploited to induce sparsity and, simultaneously, identify the subset of weights which can share the same features.

Network Pruning is arguably one of the most common technique to compress Neural Network: in a nutshell it aims at removing a set of weights according to a certain criterion (magnitude, importance, etc). Chang et al.^15^ proposed an iterative pruning algorithm that exploits a continuously differentiable version of the $\ell _{\frac{1}{2}}$ norm, as a penalty term. Molchanov et al.^16^ focused on pruning convolutional filters, so as to achieve better inference performances (with a modest impact on the recorded accuracy) in a transfer leaning scenario. Starting from a network fine-tuned on the target task, they proposed an iterative algorithm made up of three main parts: (1) assessing the importance of each convolutional filter on the final performance via a Taylor expansion, (2) removing the less informative filters and (3) re-training the remaining filters, on the target task. Inspired by the pioneering work in^7^, Pau de Jorge et al.^17^ proved that pruning at initialization leads to a significant performance degradation, after a certain pruning threshold. In order to overcome this limitation they proposed two different methods that enable an initially trimmed weight to be reconsidered during the subsequent training stages.

Knowledge Distillation is yet another technique, firstly proposed by Hinton et al.^18^. In its simplest version Knowledge Distillation is implemented by combining two objective functions. The first accounts for the discrepancy between the predicted and true labels. The second is the cross-entropy between the output produced by the examined network and that obtained by running a (generally more powerful) trained model. In^19^ Polino et al. proposed two approaches to mix distillation and quantization (see below): the first method uses the distillation during the training of the so called student network under a fixed quantization scheme while the second exploits a network (termed the teacher network) to directly optimize the quantization. Mirzadeh et al.^20^ analyzed the regime in which knowledge distillation can be properly leveraged. They discovered that the representation power gap of the two networks (teacher and student) should be bounded for the method to yield beneficial effects. To resolve this problem, they inserted an intermediate network (the assistant), which sits in between the teacher and the student, when their associated gap is too large.

Matrix Decomposition is a technique that remove redundancies in the parameters by the means of a tensor/matrix decomposition. Masana et al.^21^ proposed a matrix decomposition method for transfer learning scenario. They showed that decomposing a matrix taking into account the activation outperforms the approaches that solely rely on the weights. In^22^, Novikov et al. proposed to replace the dense layer with its Tensor-Train representation^23^. Yu et al.^24^ introduced a unified framework, integrating the low-rank and sparse decomposition of weight matrices with the feature map reconstructions.

Quantization, as also mentioned above, aims at lowering the number of bits used to represent any given parameter of the network. Stock et al.^25^ defined an algorithm that quantize the model by minimizing the reconstruction error for inputs sampled from the training set distribution. The same authors also claimed that their proposed method is particularly suited for compressing residual network architectures and that the compressed model proves very efficient when run on CPU. In Banner et al.^26^ a practical 4-bit post-training quantization approach was introduced and tested. Moreover, a method to reduce network complexity based on node-pruning was presented by He et al. in^27^. Once the network has been trained, nodes are classified by means of a node importance function and then removed or retained depending on their score. The authors proposed three different node ranking functions: entropy, output-weights norm (onorm) and input-weights norm (inorm). In particular, the input-weights norm function is defined as the sum of the absolute values of the incoming connections weights. As we will see this latter defines the benchmark model that we shall employ to challenge the performance of the trimming strategy here proposed. Finally, it is worth mentioning the Conditional Computation methods^28–30^: the aim is to dynamically skip part of the network according to the provided input so as to reduce the computational burden.

Summing up, pruning techniques exist which primarily pursue the goal of enforcing a sparsification by cutting links from the trained neural network and have been reviewed above. In contrast with them, the idea of our method is to a posteriori identify the nodes of the trained network which prove unessential for a proper functioning of the device and cut them out from ensemble made of active units. This yields a more compact neural network, in terms of composing neurons, with unaltered classification performance. The method relies on the spectral learning^5,6^ and exploits the fact that eigenvalues are credible parameters to gauge the importance of a given node among those composing the destination layer. In short, our aim is to make the network more compact by removing nodes classified as unimportant, according to a suitable spectral rating.

## Results

In order to assess the effectiveness of the eigenvalues as a marker of the node’s importance (and hence as a potential target for a cogent pruning procedure) we will consider a fully connected feed-forward architecture. Applications of the explored methods will be reported for $\ell =3$ and $\ell >3$ configurations. The nodes that compose the hidden layers are the target of the implemented pruning strategies. As we shall prove, it is possible to get rid of the vast majority of nodes without reflecting in a sensible decrease in the test accuracy, if the filter, either in its pre- or post-training versions, relies on the eigenvalues ranking. Moreover, it is also important to stress that, in general terms, the pruning of unessential nodes improves the computational efficiency of the network. As a matter of fact, reducing the number of output nodes leads a compression in terms of both memory and inference time which is directly proportional to the number of removed elements. As an example, by pruning a fraction $\alpha \;\; (< 1)$ of the total nodes, we obtain a new layer with $\alpha \cdot N$ less neurons and a memory reduction of $\alpha \cdot N$ times the number of input features.

For our test, we used three different datasets of images. The first is the renowned MNIST database of handwritten digits^31^, composed by greyscale images of dimension $28\times 28$ pixels. The second is Fashion-MNIST (F-MNIST)^32^ (an image dataset of Zalando’s items) which are still depicted with a greyscale with dimension $28\times 28$ but display an enhanced degree of inherent complexity for what concerns the type of classification requiredas compared to the basic MNIST benchmark model (more complex shapes, patterns on items). The last one is CIFAR-10^33^ a richer dataset composed by $32\times 32$ RGB images of complex real-world objects divided in 10 classes. In the main text we report our findings for Fashion-MNIST. Analogous investigations carried out for MNIST and CIFAR10 will be reported as Supplementary Information. Further, different activation functions have been employed to evaluate the performance of the methods. In the main body of the paper, we will show the results obtained for the ELU. The conclusion obtained when operating with the ReLU and $\tanh$ are discussed in the annexed Supplementary Information. In the following we will report into two separate sub-sections the results pertaining to either the single or multiple hidden layers settings.

### Single hidden layer ($\ell =3$)

**Figure 3:**
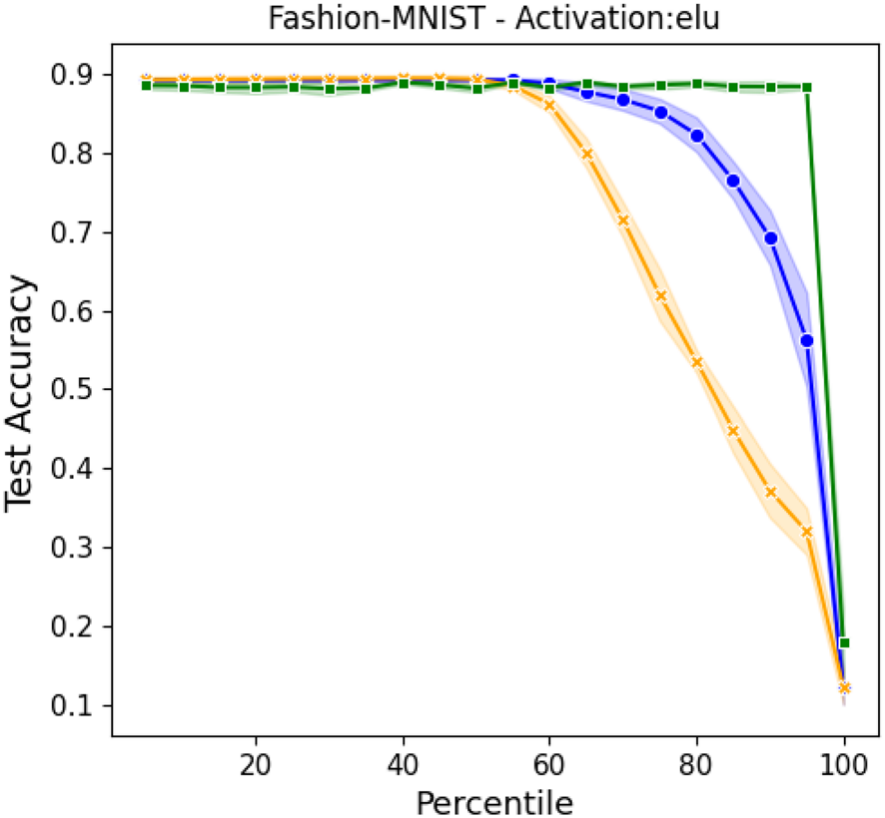
Accuracy on the Fashion-MNIST database with respect to the percentage of trimmed nodes (from the hidden layer), in a three layers feedforward architecture. Here, $N_2=500$, while $N_1=784$ and $N_3=10$, as reflecting the structural characteristics of the data. In orange the results obtained by pruning the network trained in direct space, based on the absolute value of the incoming connectivity (see main text). In blue, the results obtained when filtering the nodes after a full spectral training (post-training). The curve in green reports the accuracy of the trimmed networks generated upon application of the pre-training filter. Symbols stand for the averaged accuracy computed over 5 independent realizations. The shadowed region is traced after the associated semi-dispersion.

In Fig. 3, the performance of the inspected methods are compared for the minimal case study of a three layers network. The intermediate layer, the sole hidden layer in this configuration, is set to $N_2=500$ neurons. The accuracy of the different methods are compared, upon cutting at different percentile, following the strategies discussed in “Methods” and compared with the benchmark model (the orange profile). In the benchmark model, the neural network is trained in direct space, by adjusting the weights of each individual inter-nodes connection. Then, the absolute value of the incoming connectivity is computed and used as an importance rank of the nodes’ influence on the test accuracy (analogous to the way in which we use the eigenvalues). Such a model has been presented and discussed by He et al. in^27^. Following this assessment, nodes are progressively removed from the trained network, depending on the imposed percentile, and the ability of the trimmed network to perform the sought classification (with no further training) tested. We choose this particular type of trimming as a benchmark to our spectral pruning technique for the following reasons. First, it also amount to removing nodes from the collection, and not just sparsify the weight of the associated transfer matrices. Then, both approaches build on the concept of nodes ranking, as obtained from a suitable metric, which is respectively bound to direct vs. spectral domains. The abovementioned procedure is repeated 5 times and the mean value of the accuracy plotted in the orange curve of Fig. 3. The shaded region stands for the semi dispersion of the measurements. A significant drop of the network performance is found when removing a fraction of nodes larger than 60% from the second layer.

The blue curve Fig. 3 refers instead to the post-processing spectral pruning based on the eigenvalues and identified, as method (ii), in “Methods” section. More precisely, the three layers network is trained by simultaneously acting on the eigenvectors and the eigenvalues of the associated transfer operators, as illustrated above. The accuracy displayed by the network trained according to this procedure is virtually identical to that reported when the learning is carried out in direct space, as one can clearly appreciate by eye inspection of Fig. 3. Removing the nodes based on the magnitude their associated eigenvalues, allows one to keep stable (practically unchanged) classification performance for an intermediate layer that is compressed of about 70% of its original size. In this case the spectral pruning is operated as a post-processing filter, meaning that the neural network is only trained once, before the nodes’ removal takes eventually place.

At variance, the green curve in Fig. 3 is obtained following method (i) from “Methods” section, which can be conceptualized as a pre-training manipulation. Based on this strategy, we first train the network on the set of tunable eigenvalues, than reduce its size by performing a compression that reflects the ranking of the optimized eigenvalues and then train again the obtained network by acting uniquely on the ensemble of residual eigenvectors. The results reported in Fig. 3 indicate that, following this procedure, it is indeed possible to attain astoundingly compact networks with unaltered classification abilities. Moreover, the total number of parameters that need to be tuned following this latter procedure is considerably smaller than that on which the other methods rely. This is due to the fact that only the random directions (the eigenvectors) that prove relevant for discrimination purposes (as signaled by the magnitude of their associated eigenvalues) undergoes the second step of the optimization. This method can also be seen as a similar kind of^7^. As a matter of fact, the initial training of the eigenvalues uncovers a sub-network that, once trained, obtains performances comparable to the original model. More specifically, the uncovered network can be seen as a *winning ticket*^7^. That is, a sub-network with an initialization particularly suitable for carrying out a successful training.

Next, we shall generalize the analysis to the a multi-layer setting ($\ell >3$), reaching analogous conclusions.

### Multiple hidden layers ($\ell >3$)

Quite remarkably, the results achieved in the simplified context of a single hidden layer network also apply within the framework of a multi-layers setting.

To prove this statement we set to consider a $\ell =5$ feedforward neural network with ELU activation. Here, $N_1=784$ and $N_5=10$ as reflecting the specificity of the employed dataset. The performed tests follows closely those reported above, with the notable difference that now the ranking of the eigenvalues is operated on the pool of $N_2 + N_3 + N_4$ neurons that compose the hidden bulk of the trained network. In other words, the selection of the neuron to be removed is operated after a global assessment, i.e. scanning across the full set of nodes, without any specific reference to an a priori chosen layer.

**Figure 4:**
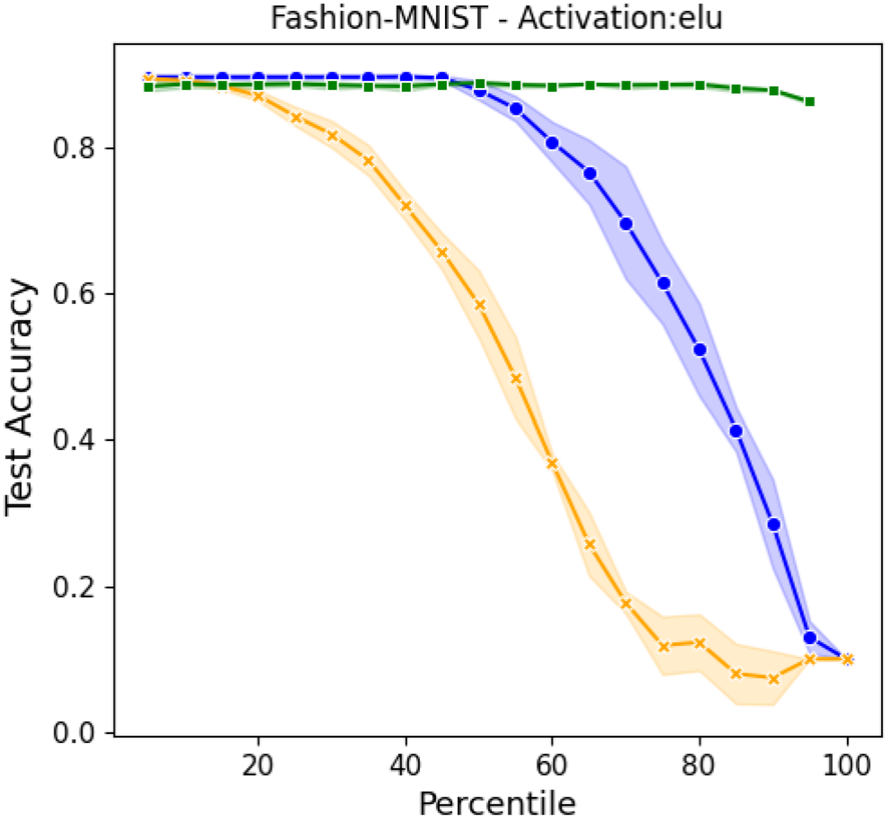
Accuracy on the Fashion-MNIST database with respect to the percentage of pruned nodes (from the hidden layers), in a five layers feedforward architecture. Here, $N_2=N_3=N_4=500$, while $N_1=784$ and $N_5=10$, as reflecting the structural characteristics of the data. Symbols and colors are chosen as in Fig. 3.

In Fig. 4, the results of the analysis are reported, assuming $N_2=N_3=N_4=500$. The conclusions are perfectly in line with those reported above for the one layer setting, except for the fact that now the improvement of the spectral pruning over the benchmark reference are even superior. The orange curve drops at percentile 20, while the blue begins its descent at about 60%. The green curve, relative to the sequential two steps training, stays stably horizontal up to about 90%.

## Conclusions

In this paper we have discussed a relevant byproduct of a spectral approach to the learning of deep neural networks. The eigenvalues of the transfer operator that connects adjacent stacks in a multi-layered architecture provide an effective measure of the nodes importance in handling the information processing. By exploiting this fact we have introduced and successfully tested two distinct procedures to yield compact networks—in terms of number of computing neurons—which perform equally well than their untrimmed original homologous. One procedure (referred as (ii) in the description) is acknowledged as a post processing method, in that it acts on a multi-layered network downstream of training. The other (referred as (i)) is based on a sequence of two nested operations. First the eigenvalues are solely trained. After the spectral pruning took place, a second step in the optimization path seeks to adjust the entries of the eigenvectors that populate a trimmed space of reduced dimensionality. The total number of trained parameters is small as compared to that involved when the pruning acts as a post processing filter. Despite that, the two steps pre-processing protocol yields compact devices which outperform those obtained with a single post-processing removal of the unessential nodes.

As a benchmark model, and for a neural network trained in direct space, we decided to rank the nodes importance based on the absolute value of the incoming connectivity. This latter appeared as the obvious choice, when aiming at gauging the local information flow in the space of the nodes, see also^27^. In principle, one could consider to diagonalizing the transfer operators as obtained after a standard approach to the training and make use of the computed eigenvalues to a posteriori sort the nodes relevance. This is however not possible as the transfer operator that links a generic layer *k* to its adjacent counterpart $k+1$, as follows the training performed in direct space, is populated only below the diagonal, with all diagonal entries identically equal zero. All associated eigenvalues are hence are zero and they provide no information on the relative importance of the nodes of layer $k+1$, at variance with what happens when the learning is carried out in the reciprocal domain.

Summing up, by reformulating the training of neural networks in spectral space, we identified a set of sensible scalars, the eigenvalues of suitable operators, that unequivocally correlate with the influence of the nodes within the collection. This observation translates in straightforward procedures to generate efficient networks that exploit a reduced number of computing units. Tests performed on different settings corroborate this conclusions. As an interesting extension, we will show in the Supplementary Information that a suitable regularization of the eigenvalues yields a general improvement of the proposed method.

## Methods

We detail here the spectral procedure to make a trained network smaller, while preserving its ability to perform classification.

To introduce the main idea of the proposed method, we make reference to formula (2) and assume the setting where $\lambda ^{(k)}_{m(j)}=0$. The information travelling from layer *k* to layer $k+1$ gets hence processed as follows: first, the activity on the departure node *j* is modulated by a multiplicative scaling factor ${\Phi }^{(k)}_{l(i), m(j)}$, specifically linked to the selected (*i*, *j*) pair. Then, all incoming (and rescaled) activities reaching the destination node *i* are summed together and further weighted via the scalar quantity $\lambda ^{(k)}_{l(i)}$. This latter eigenvalue, downstream of the training, can be hence conceived as a distinguishing feature of node *i* of layer $k+1$. Assume for the moment that ${\Phi }^{(k)}_{l(i), m(j)}$ are drawn from a given distribution and stay put during optimization. Then, every individual neuron bound to layer $k+1$ is statistically equivalent (in terms of incoming weights) to all other nodes, belonging to the very same layer. The eigenvalues $\lambda ^{(k)}_{l(i)}$ gauge therefore the relative importance of the nodes, within a given stack, and as reflecting the (randomly generated) web of local inter-layer connections (though statistically comparable). Large values of $|\lambda ^{(k)}_{l(i)}|$ suggest that node *i* on layer $k+1$ plays a central role in the economy of the neural network functioning. This is opposed to the setting when $|\lambda ^{(k)}_{l(i)}|$ is found to be small. Stated differently, the subset of trained eigenvalues provide a viable tool to rank the nodes according to their degree of importance. As such, they can be used as reference labels to make decision on the nodes that should be retained in a compressed analogue of the trained neural network, with unaltered classification performance. As empirically shown in the Results section with reference to a variegated set of applications, the sorting of the nodes based on the optimized eigenvalues turns out effective also when the eigenvectors get simultaneously trained, thus breaking, at least in principle, statistical invariance across nodes.

As we will clarify, the latter setting translates in a post-training spectral pruning strategy, whereas the former materializes in a rather efficient pre-training procedure. The non linear activation function as employed in the training scheme leaves a non trivial imprint, which has to be critically assessed.

More specifically, in carrying out the numerical experiments here reported we considered two distinct settings, as listed below:

- (i) As a first step, we will begin by considering a deep neural network made of *N* neurons organized in $\ell$ layers. The network will be initially trained by solely leveraging on the set of tunable eigenvalues. Then, we will proceed by progressively removing the neurons depending on their associated eigenvalues (as in the spirit discussed above). The trimmed network, composed by a total of $M<N$ units, still distributed in $\ell$ distinct layers, can be again trained acting now on the eigenvectors, while keeping the eigenvalues frozen to the earlier determined values. This combination of steps, which we categorize as pre-training, yields a rather compact neural network (*M* can be very small) which performs equally well than its fully trained analogue made of *N* computing nodes.
- (ii) We begin by constructing a deep neural network made of *N* neurons organized in $\ell$ layers. This latter undergoes a full spectral training, which optimizes simultaneously eigenvectors and the eigenvalues. The trained network can be compressed, by pruning the nodes which are associated to eigenvalues (see above) with magnitude smaller that a given threshold. This is indeed a post-training pruning strategy, as it acts ex post on a fully trained device.

**Figure 5:**
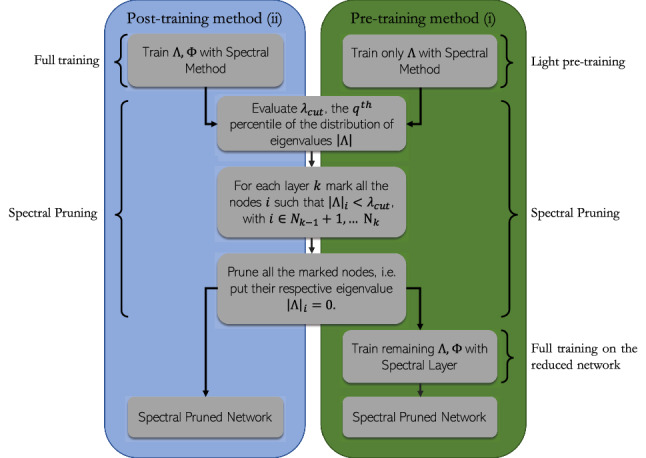
Flowchart of the pre- and post- speactral training pruning strategies as presented in “ Methods”.

To evaluate the performance of the proposed spectral pruning strategies (schematically represented in the flowchart of Fig. 5), we also introduced a reference benchmark model. This latter can be conceptualized as an immediate overturning of the methods in direct space. Simply stated, we train the neural network in the space of the nodes, by using standard approaches to the learning. Then, we classify the nodes in terms of their relevance using a proper metric to which shall make reference below, and consequently trim the nodes identified as less important. When adopting the spectral viewpoint, one can rely on the eigenvalues to rank the nodes importance. As remarked above, in fact, the eigenvalues at the receiver nodes set a local scale for the incoming activity, the larger the eigenvalue (in terms of magnitude) the more important the role played by the processing unit. As a surrogate of the eigenvalues, when anchoring the train in direct space, we can consider the quantity $\sum _{j=1}^{N_k} |w_{ij}|$, for each neuron *i* belonging to layer $k+1$, see also^27^. The absolute value prevents mutual cancellations of sensible contributions bearing opposite signs, which could incidentally hide the actual importance of the examined node.

In all explored cases, the pruning is realized by imposing a threshold on the reference indicator (be it the magnitude of the eigenvalues or the cumulated flux of incoming—and made positive—weights). Pointedly, the respective indicator is extracted for every node in the arrival layer. Then a percentile *q* is chosen and the threshold fixed to the *q*-th percentile. Nodes displaying an indicator below the chosen threshold are removed and the accuracy of the obtained (trimmed) neural network assessed on the test-set. The codes employed, as well as a notebook to reproduce our results, can be found in the public repository of this project (see Data availability section).

## Supplementary Information

Supplementary Information.

## Data Availability

All the code and data to reproduce the results can be found at https://github.com/Jamba15/SpectralTools.
