# Supplementary material for: Spectral pruning of fully connected layers
Source: Sci Rep. 2022 Jul 1;12:11201. doi: 10.1038/s41598-022-14805-7 (PMC9249877; doi:10.1038/s41598-022-14805-7)
Supplement: Supplementary file 1 — Supplementary Information. [file 41598_2022_14805_MOESM1_ESM.pdf]

# Supplementary Information of Spectral Pruning of Fully Connected Layers


**Lorenzo Buffoni[1,2,*], Enrico Civitelli[3], Lorenzo Giambagli[2], Lorenzo Chicchi[2], and Duccio Fanelli[2]**

[1]Physics of Information and Quantum Technologies Group, Instituto de Telecomunicações, Lisbon, Portugal
[2]CSDC, Department of Physics and Astronomy, University of Florence, Sesto Fiorentino, Italy
[3]LabGOL, Department of Information Engineering, University of Florence, Florence, Italy
[*]lbuffoni@lx.it.pt



## ABSTRACT

Training of neural networks can be reformulated in spectral space, by allowing eigenvalues and eigenvectors of the network to act as target of the optimization instead of the individual weights. Working in this setting, we show that the eigenvalues can be used to rank the nodes' importance within the ensemble. Indeed, we will prove that sorting the nodes based on their associated eigenvalues, enables effective pre- and post-processing pruning strategies to yield massively compacted networks (in terms of the number of composing neurons) with virtually unchanged performance. The proposed methods are tested for different architectures, with just a single or multiple hidden layers, and against distinct classification tasks of general interest.


## Analytical characterisation of inter-nodes weights in direct space

In the following, we will derive Eq. 2 in the main body of the paper. We begin by recalling that $\boldsymbol{A}^{(k)}$ is a $N \times N$ matrix. From $\boldsymbol{A}^{(k)}$ we select a square sub-block of size $(N_k + N_{k+1}) \times (N_k + N_{k+1})$, formed by the elements $\boldsymbol{A}^{(k)}_{i',j'}$ with $i' = \sum_{s=1}^{k-1} N_s + i$ and $j' = \sum_{s=1}^{k-1} N_s + j$, with $i = 1, ..., N_k + N_{k+1}$, $j = 1, ..., N_k + N_{k+1}$. We use $\boldsymbol{A}^{(k)}$ to identify the obtained matrix and proceed in analogy for $\boldsymbol{\Lambda}^{(k)}$ and $\boldsymbol{\Phi}^{(k)}$. Then:

$$
\begin{aligned}
A^{(k)}_{ij} &= \left[\boldsymbol{\Phi}^{(k)} \boldsymbol{\Lambda}^{(k)} \left(2I - \boldsymbol{\Phi}^{(k)}\right)\right]_{ij} \\
&= \left[2\boldsymbol{\Phi}^{(k)} \boldsymbol{\Lambda}^{(k)}\right]_{ij} - \left[\boldsymbol{\Phi}^{(k)} \boldsymbol{\Lambda}^{(k)} \boldsymbol{\Phi}^{(k)}\right]_{ij} \\
&= \alpha^{(k)}_{ij} - \beta^{(k)}_{ij}
\end{aligned}
\tag{1}
$$

From hereon, we will omit the apex $(k)$. Assume $\lambda_1 \dots \lambda_{N_k+N_{k+1}}$ to identify the eigenvalues of the transfer operator $\boldsymbol{A}$, namely the diagonal entries of $\Lambda$. Hence, $\Lambda_{ij} = \sum_{j=1}^{N_k+N_{k+1}} \delta_{ij} \lambda_j$.
The quantities $\alpha_{ij}$ and $\beta_{ij}$ read:

$$
\begin{aligned}
\alpha_{ij} &= 2 \sum_{k=1}^{N_k+N_{k+1}} \Phi_{ik} \lambda_k \delta_{kj} = 2\Phi_{ij} \lambda_j \\
\beta_{ij} &= \sum_{k,m=1}^{N_k+N_{k+1}} \Phi_{ik} \lambda_k \delta_{km} \Phi_{mj} \\
&= \sum_{m \in \mathscr{I} \cup \mathscr{J}} \delta_{im} \lambda_m \Phi_{mj}
\end{aligned}
$$

where $j \in \mathscr{J} = (1, ..., N_k)$ refer to the nodes at the departure layer $(k)$, whereas $i \in \mathscr{I} = (N_k + 1, ..., N_k + N_{k+1})$ stand for those at arrival. Hence, $\mathscr{I} \cup \mathscr{J} = [1, ..., N_k + N_{k+1}]$. The above expression for $\beta_{ij}$ can be further manipulated to eventually yield

$$
\begin{aligned}
\beta_{ij} &= \sum_{m \in J} \Phi_{im} \lambda_m \Phi_{mj} + \sum_{m \in I} \Phi_{im} \lambda_m \Phi_{mj} \\
&= \Phi_{ij} \lambda_j + \lambda_i \Phi_{ij}
\end{aligned}
$$

and therefore: (1) as

$$\begin{aligned} \alpha_{ij} - \beta_{ij} &= 2\Phi_{ij}\lambda_j - \Phi_{ij}\lambda_j - \lambda_i\Phi_{ij} \\ &= (\lambda_j - \lambda_i)\phi_{ij} \end{aligned} \tag{2}$$

From the above expression, one obtains the sought equation, after redefining the index $i$ to have it confined in the interval $[1,\ldots,N_{k+1}]$. By definition, the matrix of the weights, $\boldsymbol{w}$, is in fact a $N_k \times N_{k+1}$ matrix.

## MNIST and Fashion-MNIST: single hidden layer with different activation functions.

We shall here report (see Figures 1a, 1b, 1c, 2a and 2b) on the performance of the proposed trimming strategies, as applied to MNIST and Fashion-MNIST, for a single hidden layer architecture and beyond the setting reported in the main body of the paper. In particular, we will assume (i) ELU, tanh and ReLU for MNIST (ii) tanh and ReLU activation function for Fashion-MNIST (the ELU activation was employed in the main text). Here, $N_2 = 500$, while $N_1 = 784$ and $N_3 = 10$.

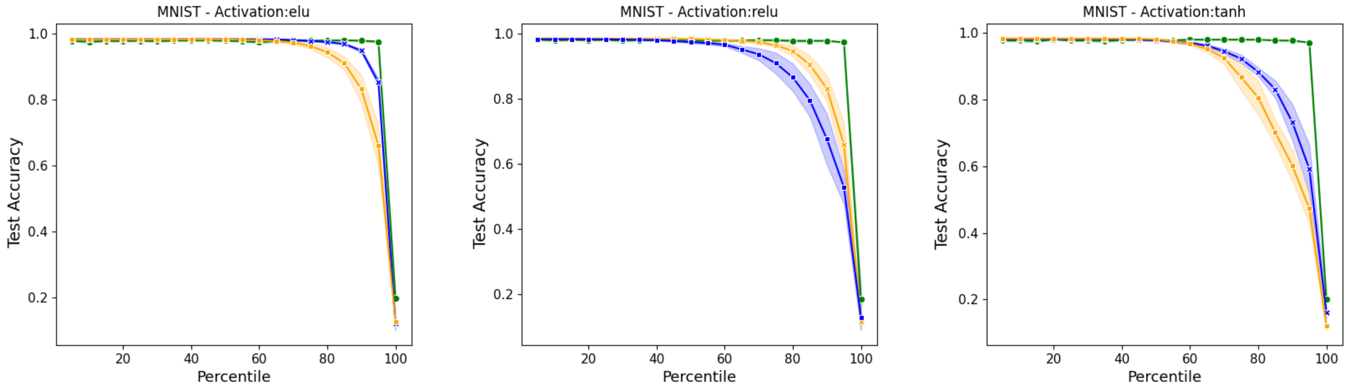


**(a)** **(b)** **(c)**

**Figure 1.** Accuracy on the MNIST database with respect to the percentage of trimmed nodes (selected from the 500 neurons that compose the sole hidden layer), in a three layers feedforward architecture. The results reported in each panel refer to a different selection of the nonlinear activation functions, respectively ELU (a), ReLU (b) and tanh (c). In orange, the results obtained by using the trimming procedure based on the absolute value of the incoming connectivity. In blue, the results obtained when filtering the nodes after a full spectral training (post-training). The curve in green displays the accuracy of the trimmed networks generated upon application of the pre-training filter. In this case, the examined network is initially trained on the set of eigenvalues, while keeping the eigenvectors frozen. After having removed unessential nodes, based on their associated eigenvalues, the network undergoes another training phase that is solely targeted to adjusting the entries of the residual eigenvectors. The shadowed region represents the semi-dispersion over 5 independent realizations. When using the Relu function, trimming on the absolute value of the incoming connectivity yields slightly better results than what found when using the post-training spectral filter. The two stages spectral trimming proves always more effective.

## MNIST and Fashion-MNIST: multiple hidden layers with different activation functions.

We will here generalize the analysis carried out in the preceding section to the case of a multilayered ($\ell > 3$) architecture (see Figures 3a, 3b, 3c, 4a and 4b). In line with the choice operated in the main body of the paper, we will assume a five layered deep neural network with $N_2 = N_3 = N_4 = 500$, and $N_1 = 784$ and $N_5 = 10$.

## Testing the trimming strategies on CIFAR10 dataset.

To assess the flexibility of the schemes outlined in Section III-B we here consider the CIFAR10 dataset and assume a modified MobileNetV2[1] adding two dense layer at the end of the network. During training we freeze all the layers, except for the two appended dense layers. These latter are trained in the spectral domain. Working in this setting, the pruning is performed on the first dense layer by using strategies both (i) and (ii), as introduced in the main body of the paper. Here again the results are compared to those obtained when using the absolute value of the incoming connectivity as an alternative trimming criterion

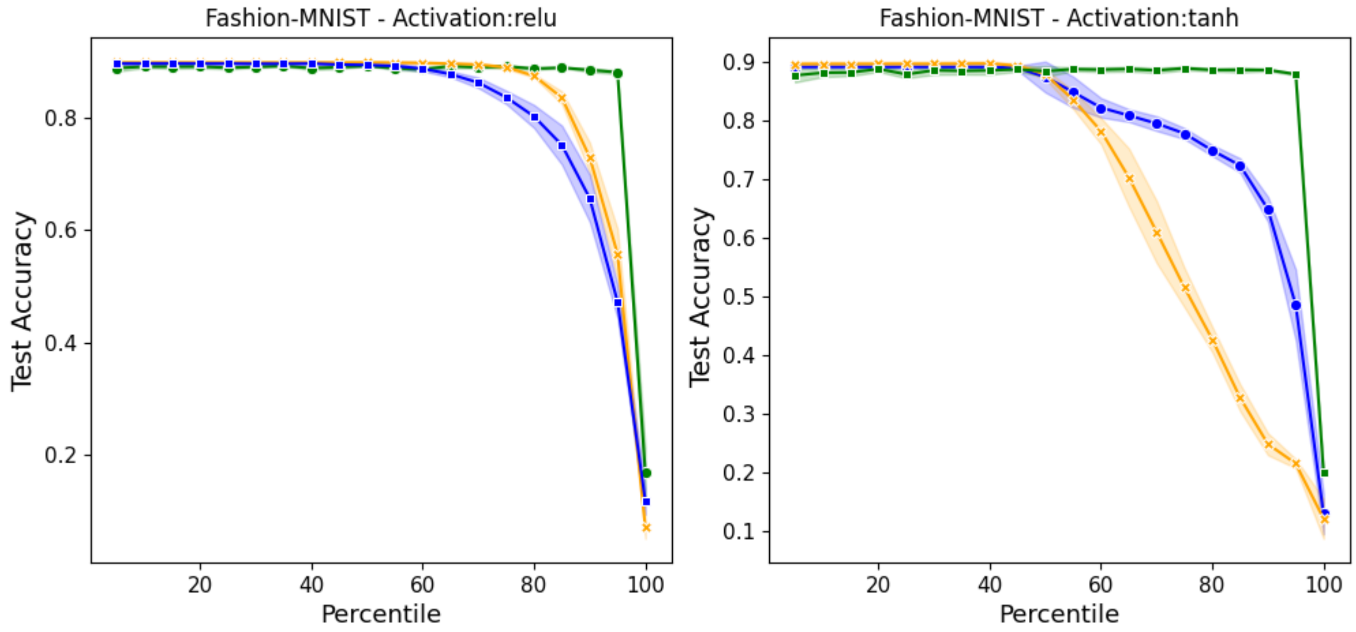


**(a)** **(b)**

**Figure 2.** Accuracy on the Fashion-MNIST database with respect to the percentage of trimmed nodes (selected from the 500 neurons that compose the sole hidden layer), in a three layers feedforward architecture. The results reported in each panel refer to a different selection of the nonlinear activation functions, respectively ReLU (b) and tanh (c). Symbols and conclusions are in line with those reported for the case of MNIST.

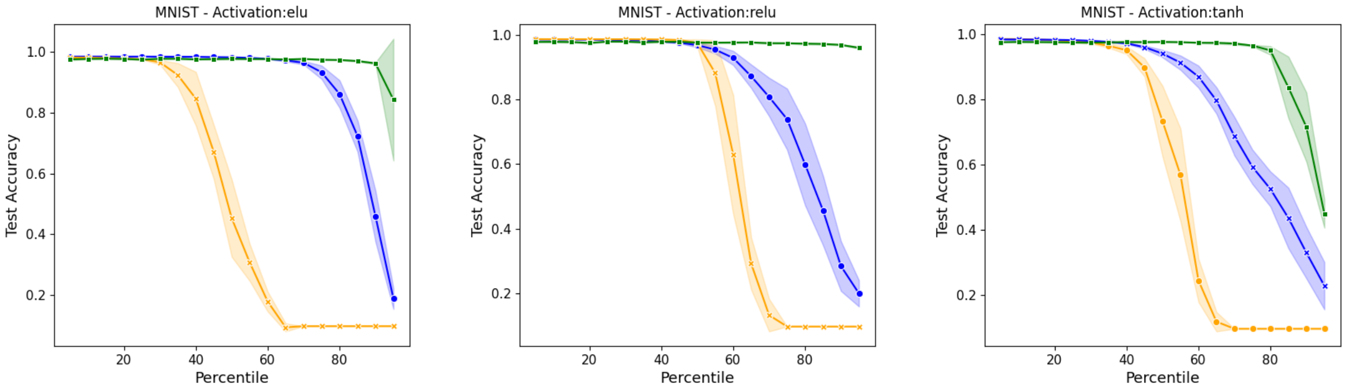


**(a)** **(b)** **(c)**

**Figure 3.** Accuracy on the MNIST database with respect to the percentage of trimmed nodes (from the set of $N_2 + N_3 + N_4$ neurons). The results in each panel refer to different choices of the non linear function, ELU (a), ReLU (b) and tanh (c). Symbols are chosen as for the case of the single hidden layer setting. It should be remarked that the spectral trimming strategies proves definitely more effective than the benchmark model anchored to direct space, also when the Relu function is employed, in the case of multiple hidden layers.

(see Figures 5a, 5b and 5c). As a further step in the analysis, we also introduce and test a $\ell_1$-norm regularization acting on the eigenvalues, so as to induce a sparse solution[2]. All experiments are performed by using a MobileNetV2 based architecture. The first dense layer is made of 512 nodes with an ELU activation function (others activations functions yield analogous results). The following regularization loss functions are considered depending on whether the training takes place in the reciprocal (spectral layer) or direct space:

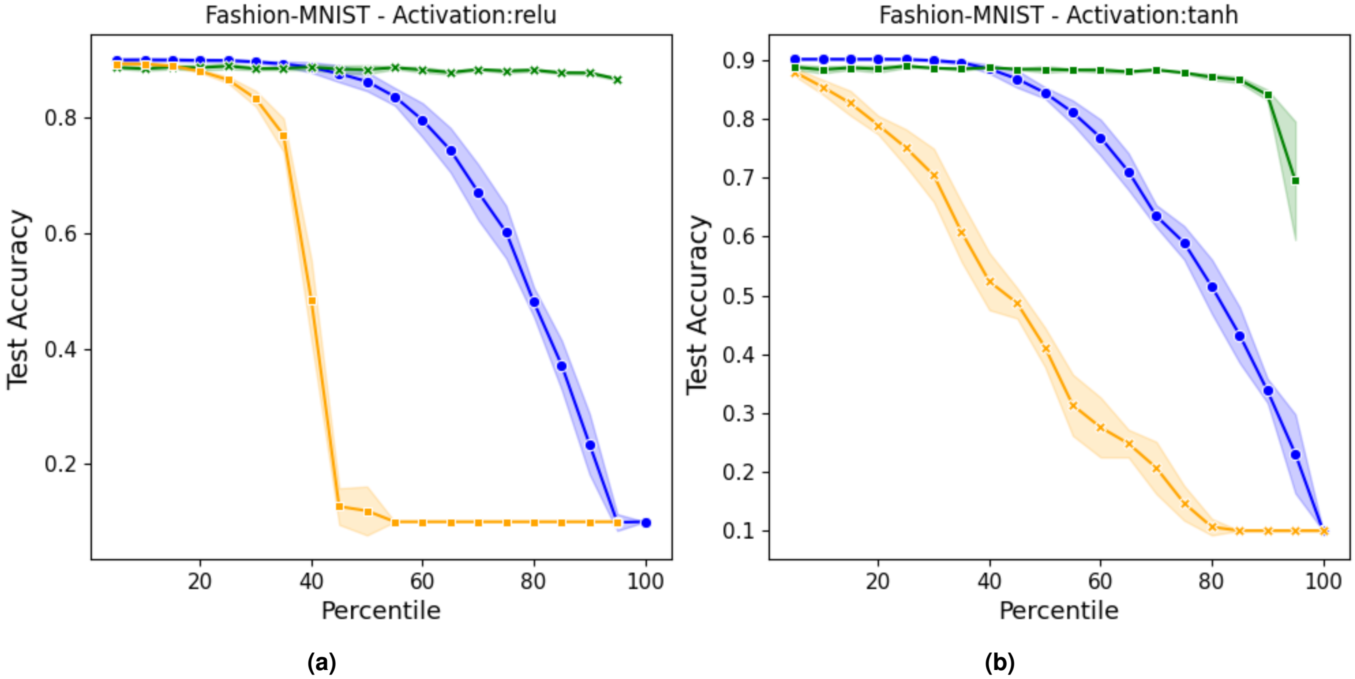


**Figure 4.** Accuracy on the Fashion-MNIST database with respect to the percentage of trimmed nodes (from the set of $N_2 + N_3 + N_4$ neurons). The results in each panel refer to different choices of the non linear activation function, ReLU (a) and tanh (b). For the symbols, see the caption of the Figures above. Also in this case the spectral filters prove always superior.

- Spectral regularization

$$L_r^{\text{spec}} = \gamma * \sum_{i=1}^{N_{\ell-1}} |\lambda_i^{(\ell-1)}|$$

- Connectivity regularization

$$L_r^{\text{conn}} = \gamma * \sum_{i,j} |w_{ij}^{(\ell-1)}|$$

where $\gamma$ stands for a suitable regularizer weight.
Clearly $L_r^{\text{conn}}$ is equivalent to a regularization which acts on the incoming absolute connectivity. In fact, $|\sum_i |x_i|| = \sum_i |x_i|$.
The $\ell_1$ regularization impacts significantly on the classification accuracy, as it can be clearly appreciated by direct inspection of Figure 6.
Choosing the correct regularizer weight ($\gamma$), the performance of the network are stable across various range of pruning thresholds, even at the highest percentile.

## References


1. Sandler, M., Howard, A., Zhu, M., Zhmoginov, A. & Chen, L.-C. Mobilenetv2: Inverted residuals and linear bottlenecks. In *Proceedings of the IEEE Conference on Computer Vision and Pattern Recognition (CVPR)* (2018).
2. Bach, F., Jenatton, R., Mairal, J. & Obozinski, G. Optimization with sparsity-inducing penalties. *Foundations Trends Mach. Learn.* **4**, 1–106, DOI: 10.1561/2200000015 (2012).

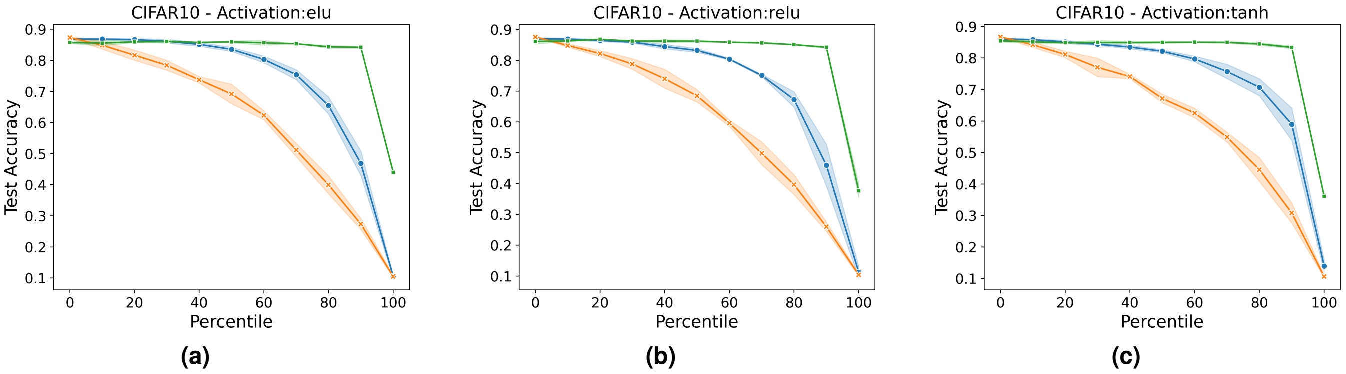


**Figure 5.** Accuracy on the CIFAR10 database with respect to the percentage of trimmed nodes (from the $\ell-1$ layer). The results in each panel refer to different non linear functions, respectively ELU (a), ReLU (b) and tanh (c). Symbols are chosen in analogy with the above (the result drawn in green are based on two different runs).

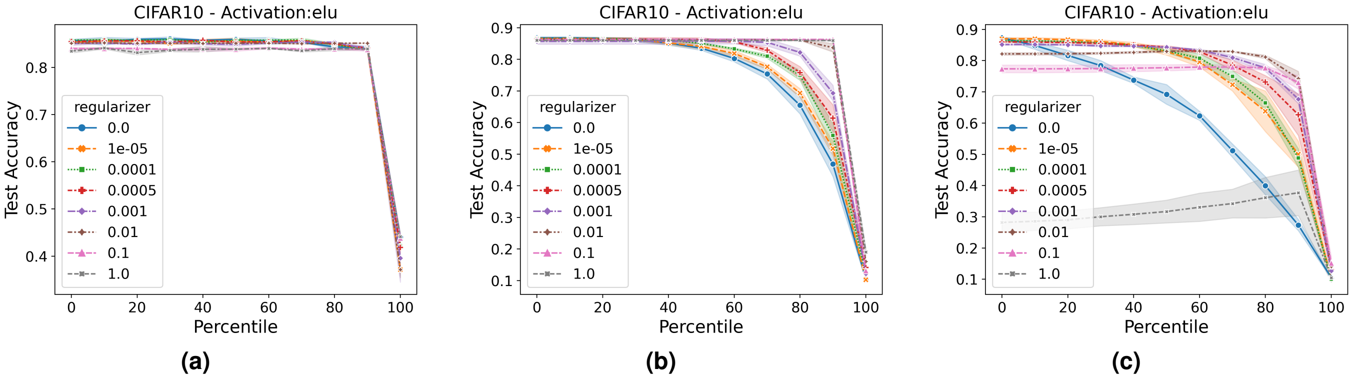


**Figure 6.** Computed accuracy on the CIFAR10 dataset against the percentage of trimmed nodes (from the first of the two dense layers appended to the MobileNet-like architecture). The panels displays the performance of the network as according to each trimming procedure, and using weights ($W$) for the $\ell_1$ regularizer. In panel (a) and (b) pre-training (based on two runs) and post-spectral filter, respectively; in panel (c) the reduction schem based on the absolute connectivity.
